# Rational Design of Phase-Engineered WS_2_/WSe_2_ Heterostructures by Low-Temperature Plasma-Assisted Sulfurization and Selenization toward Enhanced HER Performance

**DOI:** 10.1021/acsami.4c03513

**Published:** 2024-06-11

**Authors:** Bushra Rehman, K.M.M.D.K. Kimbulapitiya, Manisha Date, Chieh-Ting Chen, Ruei-Hong Cyu, Yu-Ren Peng, Mayur Chaudhary, Feng-Chuan Chuang, Yu-Lun Chueh

**Affiliations:** † Department of Materials Science and Engineering, National Tsing Hua University, Hsinchu 30013, Taiwan; ‡ College of Semiconductor Research, National Tsing Hua University, Hsinchu 30013, Taiwan; § Department of Physics, National Sun Yat-Sen University, Kaohsiung 80424, Taiwan; ∥ Department of Materials Science and Engineering, Korea University, Seoul 02841, Republic of Korea

**Keywords:** hydrogen evolution reaction, transition metal dichalcogenides, plasma-assisted chemical vapor reaction, heterostructure, WS_2_/WSe_2_ heterostructure

## Abstract

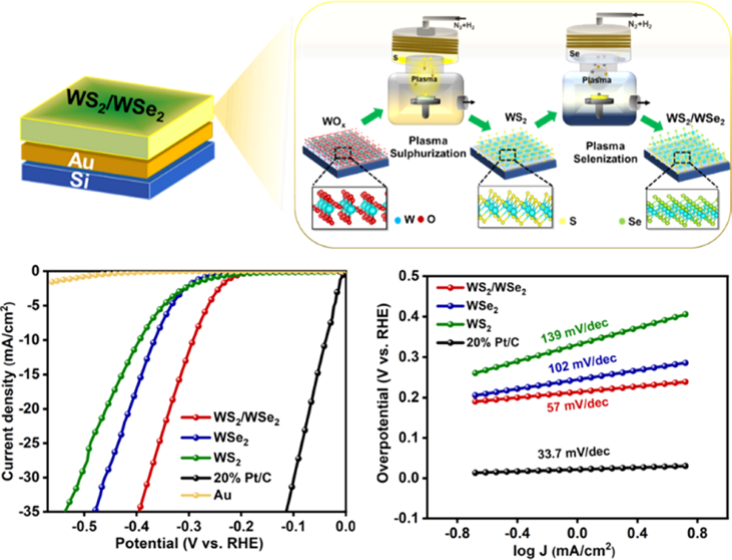

Efficient hydrogen generation from water splitting underpins chemistry to realize hydrogen economy. The electrocatalytic activity can be effectively modified by two-dimensional (2D) heterostructures, which offer great flexibility. Furthermore, they are useful in enhancing the exposure of the active sites for the hydrogen evolution reaction. Although the 1T-metallic phase of the transition metal dichalcogenides (TMDs) is important for the hydrogen evolution reaction (HER) catalyst, its practical application has not yet been much utilized because of the lack of stability of the 1T phase. Here, we introduce a novel approach to create a 1T-WS_2_/1T-WSe_2_ heterostructure using a low-temperature plasma-assisted chemical vapor reaction (PACVR), namely plasma-assisted sulfurization and plasma-assisted selenization processes. This heterostructure exhibits superior electrocatalytic performance due to the presence of the metallic 1T phase and the beneficial synergistic effect at the interface, which is attributed to the transfer of electrons from the underlying WS_2_ layer to the overlying WSe_2_ layer. The WS_2_/WSe_2_ heterostructure catalyst demonstrates remarkable performance in the HER as evidenced by its small Tafel slope of 57 mV dec^–1^ and exceptional durability. The usage of plasma helps in replacing the top S atoms with Se atoms, and this ion bombardment also increases the roughness of the thin film, thus adding another factor to enhance the HER performance. This plasma-synthesized low-temperature metallic-phase heterostructure brings out a novel method for the discovery of other catalysts.

## Introduction

Hydrogen is a key, eco-friendly alternative to fossil fuels, playing a vital role in stabilizing renewable energy supply and demand, enhancing efficiency, and increasing accessibility.^1−3^ However, the major challenge in hydrogen production is the absence of cost-effective and efficient methods. Electrochemical water splitting, particularly the hydrogen evolution reaction (HER), emerges as a promising eco-friendly solution.^4−6^ Nevertheless, a suitable catalyst is necessary to enhance the production of H_2_ by reducing the thermodynamic barrier and accelerating the overall hydrogen evolution reaction. An ideal electrocatalyst should have a substantial surface area with abundant active sites, facilitating efficient adsorption and desorption. A low or near-zero free-energy adsorption value for hydrogen (Δ*G*_H*_) is considered optimal. While platinum (Pt) is the most effective electrocatalyst, its scarcity and cost present challenges for mass production^7,8^ Thus, there is a need to discover an alternative material with high abundance, low cost, and high catalytic performance to serve as a catalyst.

Recently, two-dimensional (2D) materials, specifically transition metal dichalcogenides (TMDs),^9−13^ have brought enormous consideration because of their outstanding chemical, mechanical, electrical, and optoelectronic properties.^14,15^ The formation of large active areas in 2D layered structures leads to wide edges, which are the key active facets for better HER performance.^16,17^ 2D TMDs such as WS_2_, MoS_2_, MoSe_2_, WSe_2_, and so on, are recognized for their potential as effective electrocatalyst materials. This is attributed to their adjustable band gap, superior electrochemical properties, and cost-effectiveness.^18−21^ Recent research highlights 2D WS_2_ as a promising platinum (Pt) alternative in electrocatalysis, comparable to MoS_2_. Notably, the high specific area of WS_2_ provides abundant active sites, positioning it as a noteworthy candidate for catalytic applications. Its superior electrical conductivity, cost-effectiveness, and corrosion resistance further enhance its potential, suggesting competitiveness with Pt and other TMDs in catalytic efficiency.^22^ To address the limitations in WS_2_ as a water-splitting catalyst, various techniques, including phase engineering, defect engineering, chemical doping, heterostructure creation, and morphology modulation, have been employed to enhance charge transfer efficiency and mitigate rapid recombination of produced electrons and holes in the HER.^23−26^ Creating heterostructures is a key method to overcome WS_2_ catalyst limitations by achieving electronic transformation, thereby enhancing efficiency in energy conversion and increasing active surface sites.^27^ Various WS_2_-based heterostructures, including MoS_2_, CoSe_2_, and WSe_2_, have been studied to improve the performance of WS_2_ as an electrocatalyst for HER. For instance, Vikraman et al. synthesized the MoS_2_/WS_2_ heterostructure using chemical bath deposition and sputtering, achieving an overpotential of 129 mV and a Tafel slope of 72 mV dec^–1^.^28^ Another study by Hussain and co-workers reported the synthesis of a CoSe_2_/WS_2_ heterostructure with an onset potential of 95 mV and a Tafel slope of 44 mV dec^–1^.^29^ Recently, Sun et al. reported the one-pot solvothermal formation of WS_2_/WSe_2_ heterostructures.^30^ The fabricated catalyst on a carbon fiber paper (CFP) exhibited good HER performance with a Tafel slope of 74.08 mV dec^–1^. Here, WSe_2_ was selected for heterostructure formation with WS_2_ due to its ease of fabrication and superior electrochemical performance. Theoretical considerations indicate that Se facets in WSe_2_, with their low Gibbs free energy, play a pivotal role in H_2_ adsorption, making them excellent electrocatalysts for the HER.^31−33^

Several heterostructures have been fabricated through various methods, including solvothermal, hydrothermal, epitaxial growth, chemical vapor deposition (CVD), stacking, and exfoliation.^28−30^ A recent technique, the Laser planting strategy, involves utilizing laser pulses to generate metal single atoms (SAs) on substrates, particularly for their application as electrocatalysts.^34^ However, these methods are still inconvenient and time-consuming and also require expensive precursors. CVD has drawbacks, including time-consuming synthesis at high temperatures (exceeding 600 °C), posing a risk of substrate damage. Moreover, the need for film transfer for analysis introduces inefficiencies, leading to a costly fabrication process with issues such as wrinkles and polymer residue on the surface. CVD often yields a stable 2H phase, which is semiconducting. In contrast, the 1T phase of TMDs demonstrates promising catalytic properties for the HER. In a study, Qu et al. investigated the HER activity of different phases of TMDs such as MoS_2_, MoSe_2_, WS_2_, and WSe_2_ and found that the 1T phase of these materials possessed significantly higher HER activity than their 2H counterparts.^35^ This increased activity is attributed to the presence of active sites on the edges and defects of the 1T phase, which are not present in the 2H phase. Maintaining the metastable 1T phase of TMDs under ambient conditions for a prolonged period is challenging, since this phase can easily convert into the stable 2H phase, which is more frequently observed. As a result, the creation of 1T-TMD/1T-TMD heterostructures has not been reported so far. Therefore, there is a need to develop a facile and reliable method for designing an efficient HER electrocatalyst while comprehensively understanding and optimizing the HER activity of the 1T phase of TMDs.

We present the fabrication of a 1T-WS_2_/1T-WSe_2_ heterostructure catalyst through plasma-assisted chemical vapor reactions, specifically plasma-assisted sulfurization and selenization. Inductively coupled plasma (ICP) in these processes lowers the synthesis temperature to 350 °C, forming a 1T-metallic phase. Plasma treatment aids in metal-oxide breakdown, leading to WS_2_ creation. Se ion bombardment, assisted by plasma treatment, replaces S atoms in the top layers of WS_2_ with Se atoms, forming the WS_2_/WSe_2_ heterostructure. Confirmation was achieved via Raman spectroscopy, X-ray photoelectron spectroscopy (XPS), depth-profile XPS, atomic force microscopy (AFM), X-ray reflectivity (XRR), and cross-sectional transmission electron microscopy (TEM). Substrate temperature control in plasma sulfurization and selenization systems allows the phase transition from a semiconducting 2H phase to a metallic 1T phase. Dual plasma treatment improves surface roughness, increasing the film surface area. This heterostructure exhibits excellent hydrogen evolution reaction performance with a low Tafel slope of 57 mV dec^–1^ and remarkable stability, attributed to synergistic effects from bilayer TMD formation, reducing the energy barrier.

## Results and Discussion

**Figure 1:**
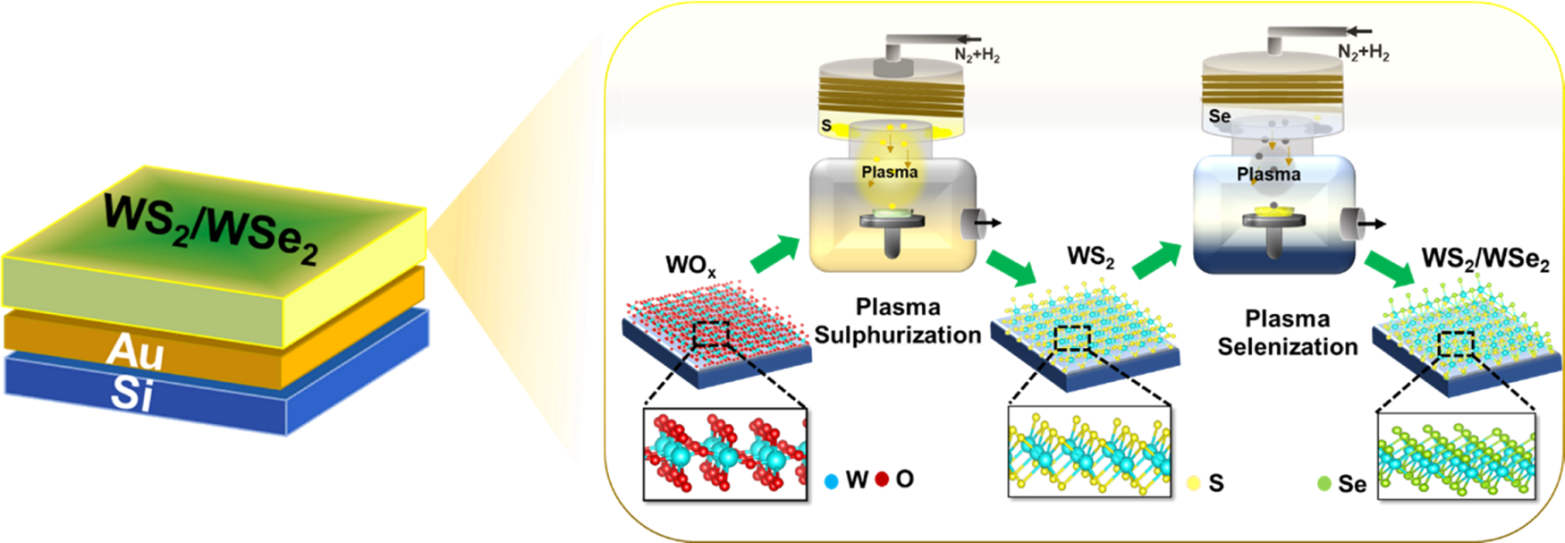
Schematic illustration depicting the formation of the WS_2_/WSe_2_ heterostructure using PACVR processes.

Figure 1 shows a schematic of the entire growth process from metal-oxide deposition to plasma treatment, resulting in the final TMD heterostructure. The electron gun deposition method was used to deposit a layer of tungsten oxide (WO_*x*_) with a thickness of 10 nm on silicon oxide substrates, followed by plasma sulfurization and plasma selenization processes, leading to the formation of the WS_2_/WSe_2_ heterostructure.

**Figure 2:**
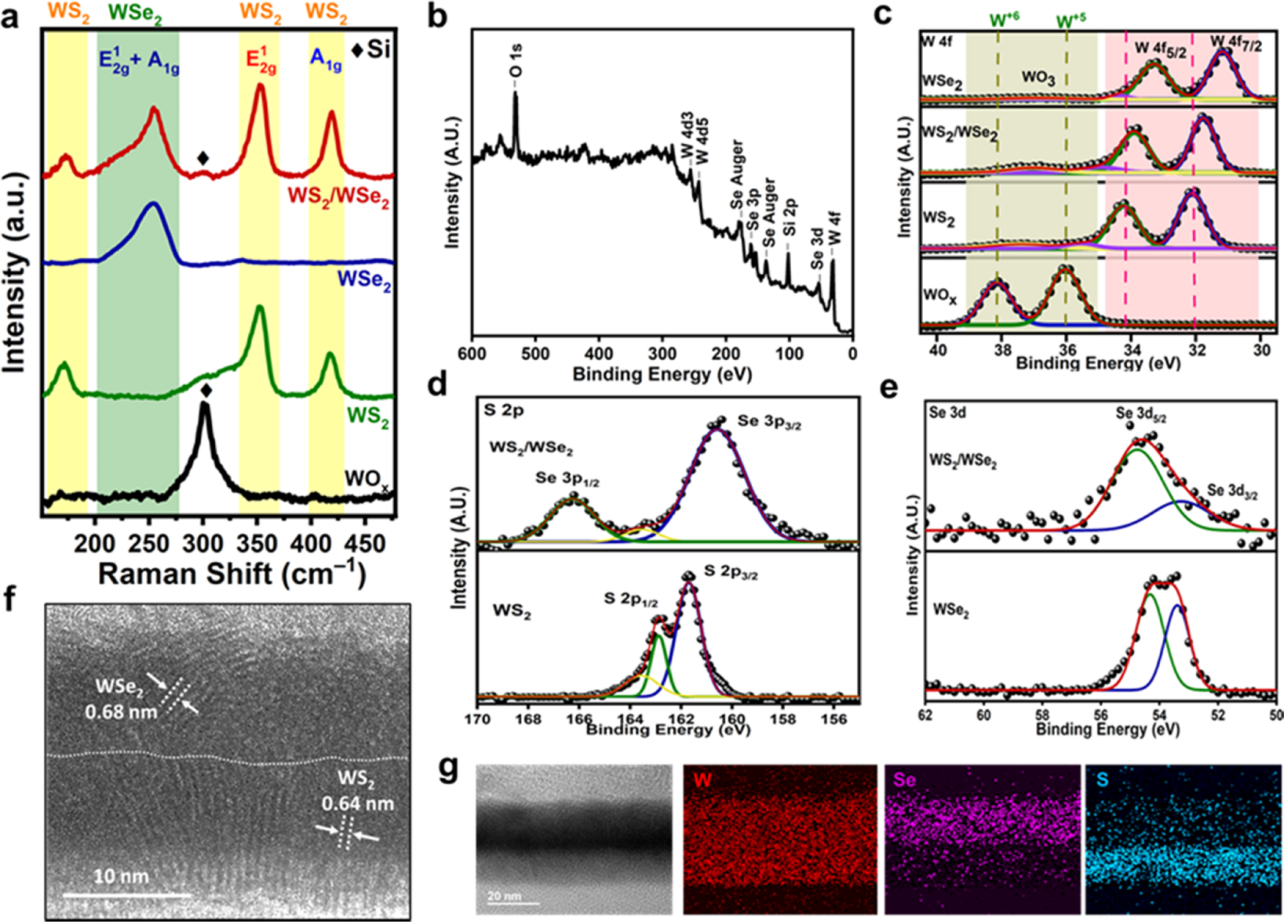
(a) Raman spectra of the WS_2_/WSe_2_ heterostructure with WO_*x*_, WS_2_, and WSe_2_. (b) Overall XPS spectrum analysis of the WS_2_/WSe_2_, (c) W 4f, (d) S 2p, and (e) Se 3d high-resolution XPS of the WS_2_/WSe_2_ catalyst. (f) Cross-sectional TEM image of the WS_2_/WSe_2_ heterostructure. (g) Elemental mapping of the WS_2_/WSe_2_ heterostructure.

The synthesis of the WS_2_/WSe_2_ heterostructure and the growth of pure WS_2_ and WSe_2_ were confirmed using Raman scattering, as shown in Figure 2a. The spectra of WO_*x*_ obtained by using the electron gun deposition method did not show any characteristic peaks, indicating its amorphous nature. However, Raman spectra of WS_2_ and WSe_2_ exhibited the characteristic peaks of in-plane W–S phonon (E_2g_^1^) and out-of-plane W–S (A_1g_) at around 352 and 417.2 cm^–1^, respectively. Additionally, a low-intensity peak at around 170 cm^–1^ confirmed the formation of the 1T phase in the WS_2_ sample.^36^ The in-plane vibration E_2g_^1^ and A_1g_ modes of W–Se bonds are closely located at 253 and 250 cm^–1^, respectively, and are merged into one peak.^37^ The presence of the characteristic WS_2_ and WSe_2_ peaks confirms the formation of the WS_2_/WSe_2_ heterostructure. A slight shift in the A_1g_ peak position of WS_2_ to a higher wavenumber for the WS_2_/WSe_2_ heterostructure strongly supports the presence of WS_2_ and WSe_2_ interaction.^38^ The results suggest that the WS_2_/WSe_2_ heterostructure synthesized using PACVR retains the distinct Raman peak positions of the individual 2D TMDs rather than exhibiting intermediate peak positions that would be expected for an alloyed WS_2*x*_Se_2(1–*x*)_ phase. Therefore, the heterostructure maintains the characteristic properties of the constituent TMDs rather than forming an alloy.^39^ The peak observed at 300 cm^–1^ is a distinctive feature of silicon originating from Si/SiO_2_ substrates. The unique characteristics of our process allow for an easy extension of the deposition technique to various substrates. To further demonstrate the versatility of PACVR, we extended the synthesis of heterostructures to other substrates, such as sapphire, as shown in Figure S1. The Raman spectra exhibited a small peak at 235 cm^–1^, which is assigned to unreacted selenium (Se) and matches the characteristic Raman shift of crystalline Se (t-Se).^40^ Additionally, we successfully synthesized the MoS_2_/MoSe_2_ heterostructure, and its formation was confirmed by the corresponding Raman spectra in Figure S2. These results highlight the wide applicability of PACVR for synthesizing various 2D heterostructures on different substrates. The characteristic modes A_1g_ at 235 cm^–1^ and E_2g_^1^ at 284 cm^–1^ originating from MoSe_2_ were evident, along with the presence of characteristic peaks for the in-plane Mo–S phonon mode, E_2g_^1^, at 380 cm^–1^ and the out-of-plane Mo–S phonon mode, A_1g_, at 405 cm^–1^, confirming the MoS_2_ formation.^41^ The presence of Raman peaks for both MoS_2_ and MoSe_2_ in the heterostructure confirms the formation of the MoS_2_/MoSe_2_ heterostructure after following the same growth process as for the WS_2_/WSe_2_ heterostructure. However, a similar drift in the A_1g_ position can be seen, as reported in the WS_2_/WSe_2_ heterostructure, confirming that there is an interaction between the two materials synthesized by PACVR. The composition, bonding characteristics, and surface electronic states of the WS_2_/WSe_2_ heterostructure were analyzed by using X-ray photoelectron spectroscopy (XPS), as illustrated in Figure 2b–e. The XPS survey spectrum of the WS_2_/WSe_2_ heterostructure (Figure 2b) confirms the presence of W, Se, and S elements, which distinguish it from those of WO_*x*_, WS_2_, and WSe_2_ (Figure S3). The W 4f spectra exhibit two dominant peaks corresponding to W 4f_7/2_ and W 4f_5/2_, indicative of W^4+^ characteristics.^30^ The doublet peaks located at 32.1 and 34.22 eV in WS_2_ and 31.1 and 33.2 eV in WSe_2_, as shown in Figure 2c, are consistent with previously reported values for WS_2_ and WSe_2_, respectively.^24,37^ The WS_2_/WSe_2_ heterostructure exhibits dominant peaks (W^4+^ 4f_7/2_ and W^4+^ 4f_5/2_) from the 1T phase at 31.7 and 33.8 eV, respectively. Interestingly, a shift in the binding energies of W 4f_7/2_ and W 4f_5/2_ for the WS_2_/WSe_2_ heterostructure suggests electron transfer from WS_2_ to WSe_2_ under the unique heterostructure effect, indicating strong electronic interaction between the two materials.^30^ The Se 3d spectra of WS_2_/WSe_2_ and WSe_2_ exhibited two well-defined peaks at 53.3 and 54.3 eV for the pure WSe_2_.^37^ The high valence peak of Se in the heterostructure shifted to a higher binding energy, confirming the strong interaction of the catalyst. Overlapping spectra of the S 2p core level with the Se 3p core level further confirmed the composition of the WS_2_/WSe_2_ heterostructure. In addition, XPS was used to confirm the successful formation of the MoS_2_/MoSe_2_ heterostructure, as shown in Figure S4. The Mo 3d spectra exhibited two peaks at 229 and 232 eV, corresponding to the binding energies of Mo 3d_5/2_ and Mo 3d_3/2_ in MoSe_2_. Similarly, the Se 3d spectra showed the corresponding 3d_5/2_ (at 54.4 eV) and 3d_3/2_ (at 55.3 eV) binding energies for Se in MoSe_2_. These results suggest that WO_*x*_ and MoO_*x*_ were effectively transformed into WS_2_/WSe_2_ and MoS_2_/MoSe_2_ heterostructures through the plasma-assisted sulfurization and selenization processes. High-resolution TEM images of the WS_2_/WSe_2_ heterostructure are shown in Figure 2f. The lattice spacings of 0.68 and 0.64 nm corresponding to the (002) facets of WSe_2_ and WS_2_ were indexed. Also, a similar kind of crystallinity is visible in the TEM images of WS_2_ and WSe_2_, suggesting that the low-temperature synthesis along with plasma could be a reason for the same (Figure S5). An amorphous deposition of WO_*x*_ is also confirmed by the TEM images of WO_*x*_. Figure 2g shows elemental mapping of the WS_2_/WSe_2_ heterostructure and reveals the presence of WSe_2_ in the top layers. However, WS_2_ appears to be near the bottom layers, confirming the conversion of the top layer into WSe_2_. Besides, energy-dispersive analysis X-ray (EDAX) spectra (Figure S6) show the equivalent presence of S and Se elements in the heterostructure.

**Figure 3:**
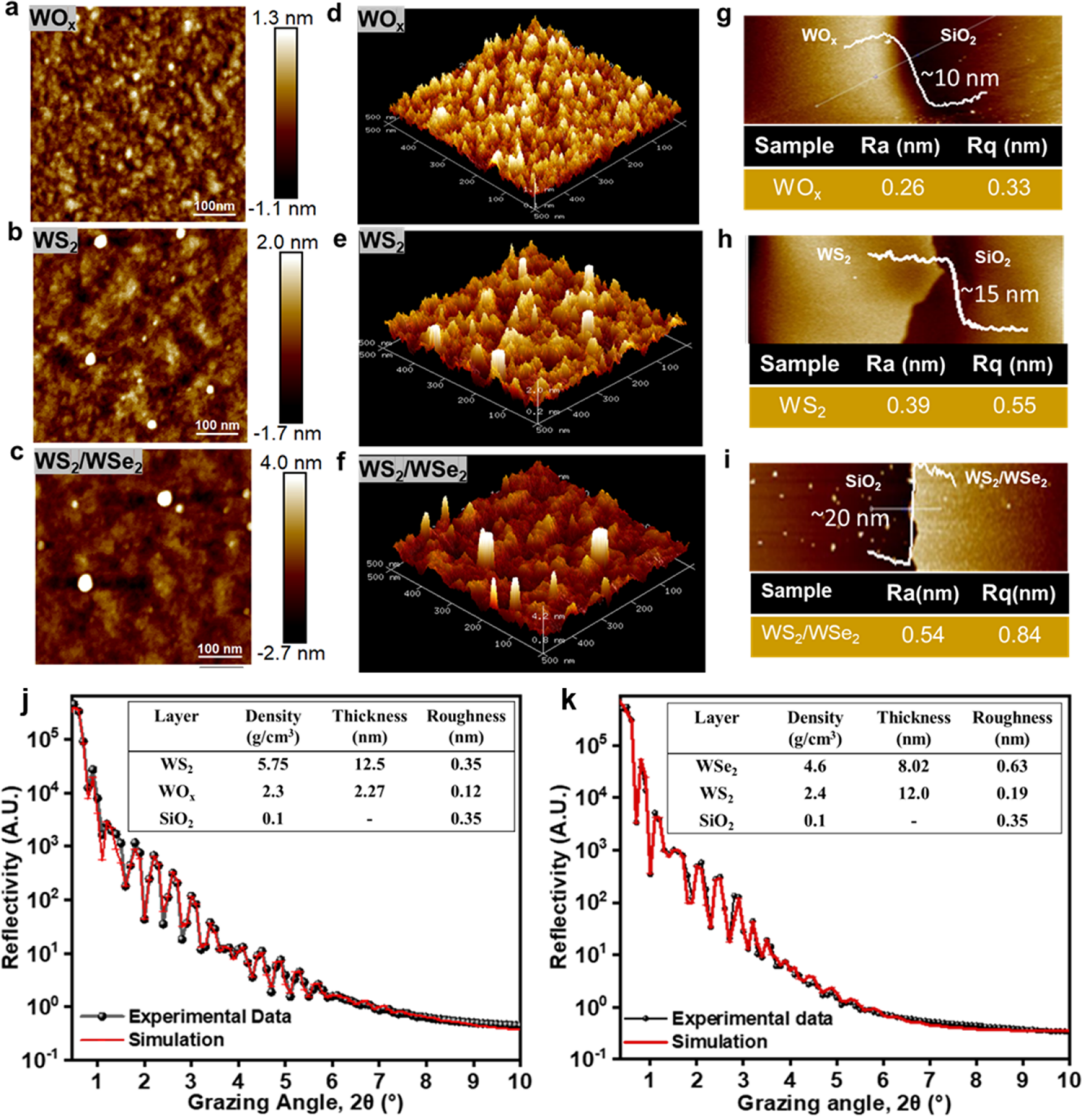
AFM images of (a) WO_*x*_, (b) WS_2_, and (c) the WS_2_/WSe_2_ heterostructure. 3D AFM images showing the surface morphology of (d) WO_*x*_, (e) WS_2_, and (f) the WS_2_/WSe_2_ heterostructure. Height profiles and corresponding surface roughness of (g) WO*_x_*, (h) WS_2_, and (i) the WS_2_/WSe_2_ heterostructure. X-ray reflectivity (XRR) measurements and simulations of (j) WS_2_ on Si substrates and (k) the WS_2_/WSe_2_ heterostructure on Si substrates.

Furthermore, atomic force microscopy (AFM) was used to study the thickness and morphology of the WS_2_/WSe_2_ heterostructure. Figure 3a–f shows 2D and three-dimensional (3D) images of as-deposited WO_*x*_, WS_2_, and WS_2_/WSe_2_ heterostructure on SiO_2_/Si substrates. It can be seen from the images that the e-gun-deposited WO_*x*_ shows a uniform granular surface with a low roughness of about 0.2 nm. However, the plasma-sulfurized WO_*x*_ film is considerably rougher than the pristine sample and exhibits prominent ridges and valleys. The root-mean-square (rms) surface roughness (σ_s_) at an area of 100 × 100 nm^2^ of the WS_2_ sample increases to ∼0.4 nm. The roughness of the samples increases with each plasma treatment given in the deposition of sulfur and selenium, thus increasing the surface area of the catalyst heterostructure film, as shown in Figure 3g–i. The surface of the WS_2_/WSe_2_ heterostructure is observed to be nonhomogeneous, with a mean square roughness of ∼0.63 nm. To evaluate the thickness of the as-grown films, AFM height profile measurements were performed along the edges. The average height of the WS_2_/WSe_2_ heterostructure film, measured from the Si substrate surface, is found to be ∼20 nm, which is double the thickness of the initially deposited film. To analyze the interface roughness and the specific thickness of the WS_2_/WSe_2_ heterostructure, X-ray reflectivity (XRR) was conducted using an eight-circle Huber diffractometer at the wiggler beamline BL17B at the National Synchrotron Radiation Research Center (NSSRC) in Taiwan. XRR allows the estimation of surface roughness, interface roughness, and layer thickness by analyzing the 2θ dependence of the reflectivity. The oscillation observed in the reflectivity is a result of the interference between X-rays reflected from the surface and interface.^45^ By analysis of the period of the oscillation, the thickness of different layers can be determined. The amplitude of the oscillation is influenced by the density difference between the film and the substrate with a larger density difference, leading to a higher amplitude. The decay rate of the reflectivity beyond the critical angle of total reflection is also indicative of surface roughness with a larger roughness, causing a higher decay rate. Figure 3j shows the reflectivity from the WS_2_-deposited silicon wafer, where the dotted and solid lines represent the experimental and simulated results, respectively. The difference between the XRR and AFM measurements is that the X-ray beam covers a larger area of 1 × 1 cm^2^ on the sample, whereas AFM probes only a local area (around 100 × 100 nm^2^).^46^ To determine the density of the WS_2_/WSe_2_ film grown on a Si wafer, XRR measurements were performed in the 2θ range 0–10°. The experimental data were fitted, revealing that the WSe_2_ film had a density of ∼4.6 g/cm^3^ and a thickness of 8.02 nm, whereas WS_2_ had a lower density of 2.4 g/cm^3^ with a thickness of 12 nm as shown in Figure 3k, resulting in a total thickness of the WS_2_/WSe_2_ heterostructure film, agreeing roughly with the result obtained by AFM. The interface roughness plays an important role in determining the adhesion between the layers. After analyzing the XRR results, the roughness of the WSe_2_ layer was determined to be 0.63 nm and the interface roughness of WS_2_ was 0.19 nm. As can be seen from the tables in the insets of Figure 3j,k, we can see that the roughness of the WS_2_ sample is lower as compared to the heterostructure, which is due to the double exposure of the sample to plasma, resulting in hills and valley formations, thus increasing the roughness of the surface. The observation that the surface roughness is inversely proportional to the density of the film suggests that the film with a lower density has a higher surface roughness, as reported in previous studies.^47^ However, this relationship might not directly apply to all interfaces or conditions, as factors like deposition process, material properties, or heterogeneity could alter this relationship at the interface of the films.^48^

The intensity of X-ray reflectivity for a rough surface is reduced by a factor of exp^–σ^2^*q*_*z*_^2^^,where σ is the root-mean-square roughness and *q*_*z*_ is the wavevector perpendicular to the sample surface. This indicates that the surface roughness affects the slope of the X-ray reflectivity curve. As shown in Figure 3j,k, the intensity decreases more rapidly with the increase in roughness. The thickness oscillations, known as “Kiessig fringes”, are clearly visible.^49^ The period of oscillations is directly related to the film’s thickness (*D*), given by , for which *t* and *D* represent the period of oscillations and film thickness, respectively. The XRR analysis may be affected by systematic errors due to even minor variations in the thickness of the film as the beam in this experimental configuration completely covers the sample. Owing to the fundamental difference in the interaction between the AFM tip and the surface compared to X-rays with the sample, the information obtained about surface roughness from AFM and XRR can be different. The surface irregularities and type of roughness play crucial roles in determining the results of AFM and XRR measurements. Typically, XRR and AFM yield similar roughness parameters for very flat surfaces with root-mean-square (rms) roughness within the range of several angstroms. However, for surfaces with high irregularities and complex height distributions, significant differences in the roughness parameters obtained by AFM and XRR can be observed.^50^ Surface roughness, determined by XRR, provides a broader view, assessing the roughness across the entire film surface of the catalyst. The results show an increase in roughness from 0.12 nm in WS_2_ to 0.63 nm in the WS_2_/WSe_2_ heterostructure, indicating the impact of dual plasma on the same surface. Additionally, interface roughness elucidates electron transfer mechanisms and adhesion, confirming the conversion and the presence of an interface, significantly influencing the HER performance.

**Figure 4:**
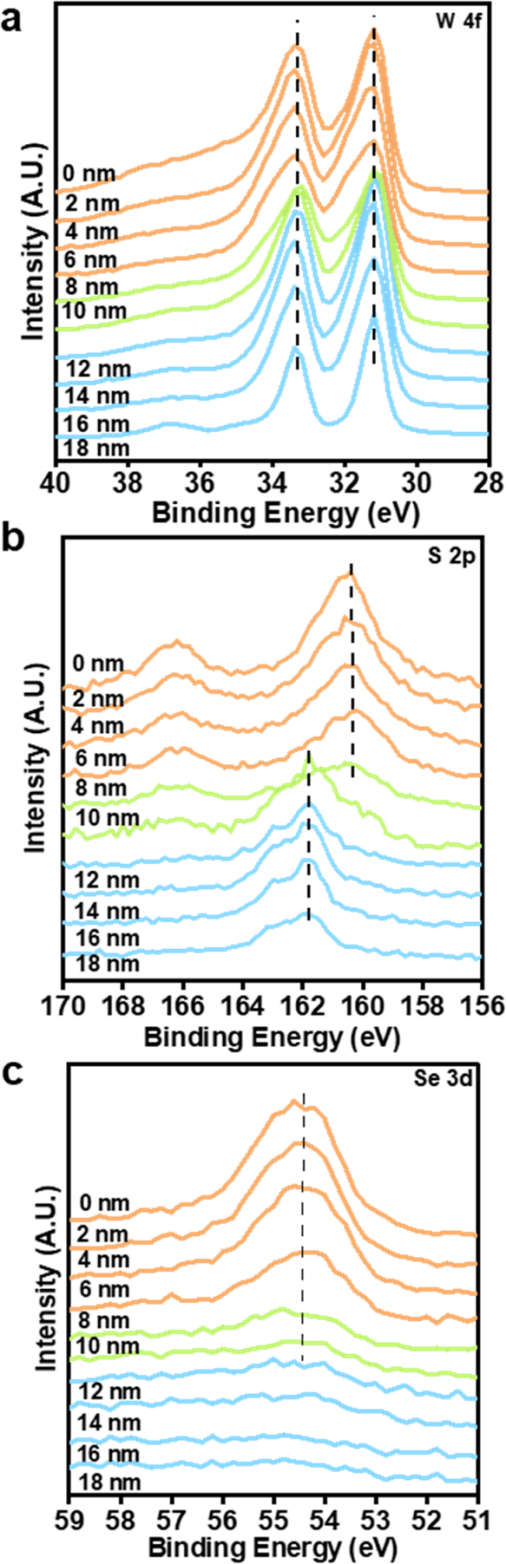
XPS spectra of (a) W 4f, (b) S 2p, and (c) Se 3d of the WS_2_/WSe_2_ heterostructure at different depth profiles.

To investigate interfacial effects in WS_2_/WSe_2_ heterostructures and to study the depth of penetration of Se ions into the WS_2_ film to form the WS_2_/WSe_2_ heterostructure, we analyzed the XPS depth profiles. Initially, the intensity of W 4f increases as we move deeper into the layers, and a similar trend is followed for the Se 3d peak. However, the peak of Se diminished after 8 nm, which indicates that the conversion of WS_2_ to WSe_2_ is possible only in the top layers (represented by the orange graph as shown in Figure 4, while the bottom 8–10 nm still corresponds to WS_2_ (depicted by the blue graph)). The S 2p spectra show an overlapping spectrum with the Se 3p core level in the upper 10 nm-thick WS_2_/WSe_2_ heterostructure, exhibiting a picture of the Se atoms knocking out the S atoms in the top layers. Quantifying selenium in the presence of sulfur using XPS presented challenges due to spectral overlap between the S 2p and Se 3p peaks (Se 3p_3/2_ and Se 3p_1/2_), along with the S 2s and Se 3s peaks. Considering the Se 3p_3/2_–3p_1/2_ doublet separation (5.8 eV) and constraining the 3p_3/2_–3p_1/2_ peaks to a 2:1 ratio,^51^ the remaining structure corresponded to the S 2p peak(s), which appeared as a minor peak visible in the fitted spectra of the WS_2_/WSe_2_ heterostructure (Figure 2d). This interface provides an electron transfer from WS_2_ to WSe_2_, thus explaining the enhanced performance of the heterostructure compared to the bare WS_2_ and WSe_2_ catalysts.

**Figure 5:**
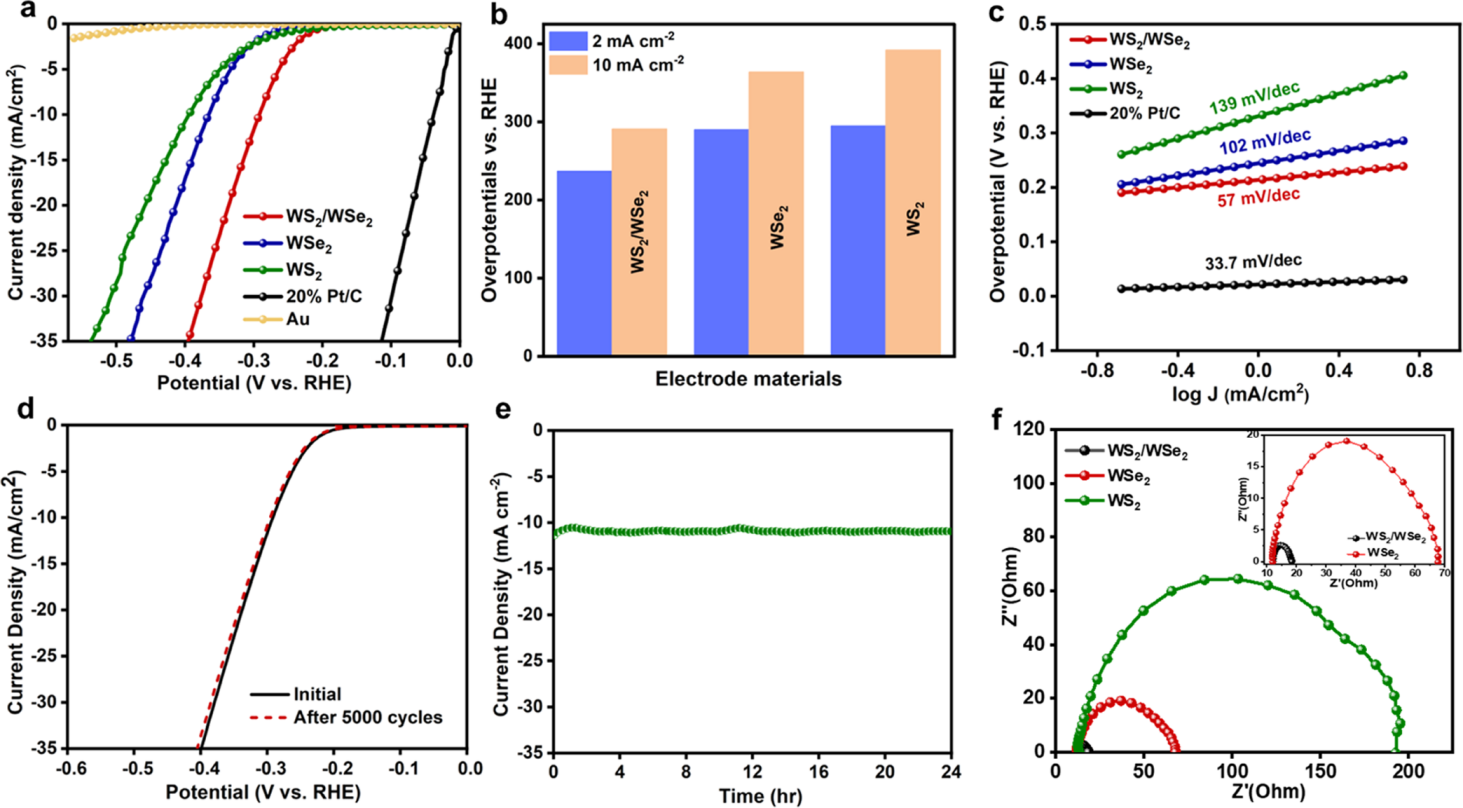
Electrocatalytic performance of different catalysts for HER in the 0.5 M H_2_SO_4_ solution: (a) polarization curves, (b) overpotentials at 2 and 10 mA cm^–2^ vs RHE, (c) Tafel plots, (d) polarization data before and after 5000 cyclic voltammetry cycles, (e) time-dependent current density (*i*–*t*) curve, and (f) Nyquist plots obtained from EIS measurements (inset: EIS profiles for WS_2_/WSe_2_).

For HER performance, a conducting surface is required. For this reason, the as-synthesized WS_2_/WSe_2_ heterostructure films were transferred onto a gold-coated silicon substrate as shown in Figure S7, where the synthesized film was separated from the Si surface using a PMMA layer as support. The PMMA/2D material film was then removed from the pristine substrate by scraping its edges with tweezers, and then it was transferred to a diluted ammonia solution (NH_4_OH/deionized (DI) water = 1:5) for separation and finally peeled off in DI water for cleaning before being transferred onto the conducting Au/Si substrate. The HER performance of the WS_2_/WSe_2_ heterostructure was evaluated using a three-electrode setup in a 0.5 M H_2_SO_4_ solution as shown in Figure S8. To facilitate comparison, the linear sweep voltammetry (LSV) curves of the as-synthesized WS_2_/WSe_2_, WS_2_, and WSe_2_, as well as a bare Au-coated silicon substrate and commercial Pt/C powders, were collected and plotted together as shown in Figure 5a. A comparison of the results showed that the Au substrate had a negligible impact on the catalytic performance, thus confirming that the HER activity observed was solely due to the as-synthesized 2D films. The ohmic drop is eliminated by performing IR correction using the resistivity (*R*_s_) value obtained from EIS curves (Table S1). Figure 5b shows a bar graph presenting a comparative study of overpotentials at 2 mA cm^–2^ and 10 mV cm^–2^, respectively, and it can be seen that the WS_2_/WSe_2_ catalyst exhibits the best performance and the lowest overpotential of 237 and 291 mV, respectively. However, bare WS_2_ and bare WSe_2_ show higher overpotentials of approximately 392 and 364 mV, respectively, under a current density of 10 mA cm^–2^. The efficient electron–hole separation resulting from the introduction of heterointerfaces leads to a significant decrease in the overpotential.^52^ Additionally, 1T phase materials exhibit a lower overpotential compared to 2H materials due to their metallic properties and lower Δ*G*_H_ at the basal plane and edge sites, which results in denser active sites than 2H phase materials^52^ It is believed that the 1T-WS_2_/1T-WSe_2_ catalyst exhibits superior electrocatalytic performance in the HER due to the diffusion of Se atoms into the top layer, replacing the S atoms. This diffusion leads to low energy barriers for orbital overlap in H* migration, known as the Volmer reaction, and H_2_ formation, resulting in better catalytic performance than those of pristine WS_2_ and WSe_2_ materials.^52^ This observation is shown by elemental mapping (Figure 2f), which reveals the presence of diffused Se atoms in upper layers; however, as we follow the spectra further, only S atoms are present. The rate-limiting stages and electrocatalyst behavior can be further understood through the Tafel plot derivative. In the case of hydrogen evolution, the reaction kinetics can be derived from a series of reactions. First, during the discharge step, hydrogen ions are adsorbed on the active sites, which is termed the Volmer reaction, as shown in eq 1. This is followed by either an electrochemical desorption step, known as the Heyrovsky reaction, as shown in eq 2, or a recombination step, known as the Tafel reaction, as shown in eq 3
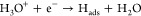
1
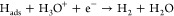
2
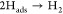
3Note that the Tafel slope is a crucial parameter used to evaluate catalyst performance and determine the rate-limiting reaction of the HER. The Volmer, Heyrovsky, and Tafel reactions can each limit the HER when the Tafel slope is around 120, 40, and 30 mV dec^–1^, respectively. To determine the rate-determining step of the HER process on the heterostructure catalyst, Tafel plot analysis was conducted, as shown in Figure 5c. The Tafel plots were obtained from the corresponding LSV curves using the Tafel equation, η = *b* log(*j*/*j*_0_), where *b*, *j*_0_, and *j* represent the Tafel slope, the exchange current density, and the current density, respectively.^53^ A Tafel slope of 31 mV dec^–1^ was confirmed for the Pt/C powders, which is consistent with the reported values. The extracted Tafel slope of WS_2_/WSe_2_ is 57 mV dec^–1^, which is smaller than those of WS_2_ (139 mV dec^–1^) and WSe_2_ (102 mV dec^–1^) (Figure 5c). The obtained results indicate that the HER process on the heterostructure catalyst occurs through Volmer–Tafel and/or Volmer–Heyrovsky reaction steps. Moreover, the low Tafel slope value suggests that the WS_2_/WSe_2_ heterostructure catalyst can generate a fast response current at a lower overpotential, indicating its superior hydrogen evolution efficiency.^30^ Compared with the recently reported values presented in Table 1, the Tafel slope of the WS_2_/WSe_2_ heterostructure as the catalyst is notably better. Thus, WS_2_/WSe_2_ heterostructures are flourishing electrocatalysts for HER. The intrinsic activity of the catalyst materials was examined by analyzing the turnover frequency (TOF). Figure S9 shows the TOF performance of the WS_2_/WSe_2_ heterostructure at different overpotentials. The heterostructure exhibits a better TOF of 2.7 s^–1^ than pure WS_2_ and WSe_2_ whose calculated values are 0.4 and 0.89 s^–1^, respectively, at an overpotential of 300 mV.

**Table 1:** Comparison of the Electrochemical Performance of TMD-Based HER Catalysts^a^

catalyst	substrate	synthesis method (morphology)	Tafel slope (mV dec^–1^)	overpotential at 10 mA cm^–2^ (mV)	*R*_ct_ (Ω)	ECSA (cm^2^)	TOF (s^–1^)	refs
WS_2_/WSe_2_ heterostructure	Au/Si	PACVR (thin film)	57	291	6.37	151.1	2.7 (η = 0.3 V)	this work
1T-WS_2_	SiO_2_/Si	PECVD (thin film)	95	347	225			(24)
1T-WSe_2_	SiO_2_/Si	sputtering (thin film)	126.3	220		5.98		(50)
Ni-WSe_2_	GCE	solvothermal (flower-like)	86	259	440	73		(33)
WS_2_–CoS_2_	CC	CVD (nanosheets)	68	119	7.12	30.8		(55)
Pd/WS_2_		hydrothermal + CVD (nanowires)	70	56	2.45		2.2 (η = 0.1 V)	(56)
MoS_2_–WSe_2_	FTO	sputtering + CVD (nanograins)	76	116	3.4	109.1		(38)
MoS_2_–WS_2_	FTO	sputtering + CVD (cauliflower-like)	72	129	2.6			(28)
NiCo-WSe_2_	CC	hydrothermal (nanoflower)	118.6	205	5	732.5	0.008 (η = 0.3 V)	(57)
MoS_2_–WS_2_	SiO_2_/Si	PECVD (thin film)	99	294	20.4			(52)
WS_2_/WSe_2_	CFP	solvothermal (flower-like)	74.08	121	10.38		0.036 (η = 0.3 V)	(30)

a CC: carbon cloth, FTO: fluorine-doped tin oxide, GCE: glassy carbon electrode, and CFP: carbon fiber paper.

Furthermore, the stability and durability of the WS_2_/WSe_2_ heterostructure as the catalyst were assessed through polarization curve measurements and Raman spectroscopy analysis after 5000 cycles. The results, as shown in Figure 5d, demonstrate that there is almost no degradation of the current density, indicating the robustness of the WS_2_/WSe_2_ heterostructure. This is attributed to the compositional and structural stability of the WS_2_/WSe_2_ heterostructure. The Raman spectra of the initial and cycled samples, as shown in Figure S10, also indicate similar peak positions, further confirming the durability of the catalyst. Additionally, Figure 5e shows that the current density remains highly stable after 24 h, supporting the stability of the WS_2_/WSe_2_ heterostructure as the catalyst. The HER kinetics were investigated using Nyquist plots obtained from electrochemical impedance spectroscopy (EIS) measurements, and each plot exhibits a similar semicircle profile without Warburg impedance in the low-frequency range, indicating rapid mass transport and a kinetically controlled reaction as shown in Figure 5f. When analyzing EIS at different overpotentials, it was found that the impedance properties were similar, suggesting that the same electrochemical processes occurred in 0.5 M H_2_SO_4_ during all tests and the equivalent circuit depicted in Figure S11 supports this finding, for which the electrochemical kinetics between the electrocatalysts and electrolytes were represented by the charge transfer resistance (*R*_ct_). Analysis in Table S1 confirms that the *R*_ct_ of the WS_2_/WSe_2_ heterostructure is significantly lower than those of WS_2_ and WSe_2_, indicating that the WS_2_/WSe_2_ heterostructure possessed a higher catalytic performance because of the faster electron exchange. The electronic structure of the heterostructure was modulated by the heterointerface between WS_2_ and WSe_2_, which boosted the charge transference and improved the catalytic activity of the WS_2_/WSe_2_ heterostructure as the catalyst. At the heterointerface between the metallic 1T materials, an overlap of d-orbitals between the WS_2_ and WSe_2_ heterostructure forms a lower energy barrier, which enhances the electrochemical activity.^52^ Additionally, the 1T phase WS_2_/WSe_2_ heterostructure can be catalytically active at both the basal plane and edge sites, leading to a high HER performance. Compared with a single WS_2_ or WSe_2_, the WS_2_/WSe_2_ heterostructure exhibits enhanced HER performance.^52^

**Figure 6:**
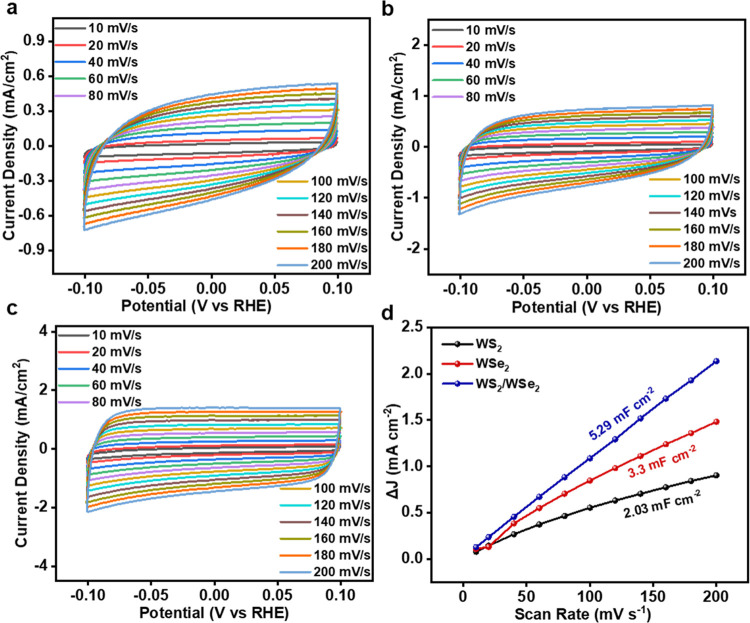
Cyclic voltammetry curves at different scan rates for (a) WS_2_, (b) WSe_2_, and (c) the WS_2_/WSe_2_ heterostructure. (d) Linear relation between the scan rate and the current density difference for the WS_2_/WSe_2_ heterostructure.

The electrochemical double-layer capacitance (*C*_dl_) was used to assess the electrocatalytically active surface area (ECSA). The value of ECSA is directly proportional to the value of *C*_dl_, which can be determined from cyclic voltammetry (CV) curves. The *C*_dl_ values for the WS_2_, WSe_2_, and WS_2_/WSe_2_ heterostructure electrocatalysts were evaluated in the non-faradaic region by conducting CV studies at scan rates ranging from 10 to 200 mV s^–1^. The results of these studies are shown in Figure 6a–c. ECSA was evaluated using the following relation: ECSA = *C*_dl_/*C*_s_, where *C*_dl_ is the double-layer capacitance and *C*_s_ is the specific capacitance. *C*_s_ = 0.035 mF cm^–2^ for 0.5 M H_2_SO_4_.^1^Table 2 includes the electrocatalytic parameters that were extracted from the plotting of different electrochemical graphs. The WS_2_/WSe_2_ heterostructure catalyst exhibited a linear slope of 5.29 mF cm^–2^, which is higher than the slopes of WS_2_ (2.03 mF cm^–2^) and WSe_2_ (3.3 mF cm^–2^), indicating that the WS_2_/WSe_2_ heterostructure has a higher exposure to efficient active sites, which contributes to its excellent HER performance as shown in Figure 6d. To account for the impact of surface topology on catalyst performance, the LSV data were adjusted by using ECSA normalization, aiming to ascertain the inherent HER activity for each catalyst (Figure S12). Normalizing the current using ECSA (Figure 5a) resulted in a shift in the order of HER activity compared to the current normalized based on the geometric surface area. The WS_2_/WSe_2_ heterostructure catalyst still exhibits the lowest onset potential compared to the pure WS_2_ and WSe_2_ catalysts.

**Table 2:** Electrocatalyst HER Measurements of WS_2_, WSe_2_, and the WS_2_/WSe_2_ Heterostructure

electrocatalyst	overpotential at 10 mA cm^–2^ (mV vs RHE)	Tafel slope (mV dec^–1^)	exchange current density *j*_o_ (mA cm^–2^)	double-layer capacitance (*C*_dl_) (mF cm^–2^)	ECSA (cm^2^)
Pt	–38	33.7	2.92		
WS_2_	–392	139	0.05	2.03	58
WSe_2_	–364	102	1.24	3.3	94.2
WS_2_/WSe_2_	–291	57	1.73	5.29	151.1

To study the performance of the WS_2_/WSe_2_ heterostructure as the electrocatalyst under alkaline conditions, the WS_2_/WSe_2_ heterostructure was tested in a 0.5 M KOH solution. However, it was not as impressive as its performance under a 0.5 M H_2_SO_4_ acidic solution, as shown in Figure S13. Nevertheless, it still outperformed WS_2_ and WSe_2_, which supports the successful combination of WS_2_ and WSe_2_. To elucidate the mechanism and assess the impact of specific sulfur-to-selenium ratios in the WS_2_/WSe_2_ heterostructure, experiments were conducted, and the corresponding results are shown in Figure S14. The WS_2_ to WSe_2_ ratio in the heterostructure was manipulated by varying the growth time, with the conversion of WS_2_ to WSe_2_ in the top layers increasing as the growth time extended from 15 to 60 min. Figure S14a illustrates the variation in WS_2_/WSe_2_ ratios with the growth time at 350 °C. The conversion ratio was confirmed by XPS, as shown in Figure S14b. In Figure S14b, the W 4f spectra were deconvoluted into specific peaks corresponding to WSe_2_ and WS_2_ in the WS_2_/WSe_2_ heterostructure. The intensity ratio of the peaks for WS_2_ (represented by yellow and magenta colors for W 4f_7/2_ and W 4f_5/2_, respectively) and WSe_2_ (represented by blue and green colors for W 4f_7/2_ and W 4f_5/2_, respectively) changes with the concentration of the two components in the heterostructure. To quantify the conversion ratio of WS_2_ to WSe_2_ in the top layers, the area under the plasma-assisted W 4f_5/2_ and W 4f_7/2_ bands (*A*_W 4f_5/2__ + *A*_W 4f_7/2__ for WSe_2_) is compared to the total area of the two components in the spectra, including WSe_2_ and WS_2_, (*A*_W 4f_5/2__ + *A*_W 4f_7/2__)_WSe_2__ + (*A*_W 4f_5/2__ + *A*_W 4f_7/2__)_WS_2__, irrespective of the presence of oxidation states of tungsten (WOx). The conversion increased from 27% for a growth time of 15 min to 73% for a growth time of 60 min. Subsequently, the impact of each component on the HER performance was evaluated using the obtained WS_2_/WSe_2_ (%) ratios. Figure S14c demonstrates that the optimal performance can be achieved with a WS_2_/WSe_2_ ratio of 53–47%, followed by the presence of 73% WSe_2_ in the heterostructure. These findings provide valuable insights into the correlation between the compositions of the heterostructure and its catalytic performance in the HER. The results indicate that the improved electrocatalytic properties of the WS_2_/WSe_2_ heterostructure can be attributed to an increase in active facets, good electron transfer, and large surface area. The outstanding HER performance of the WS_2_/WSe_2_ heterostructure can be attributed to several factors. First, the similar crystal structure and lattice constants of WS_2_ and WSe_2_ provide a foundation for forming the heterostructure at a low temperature, leading to metallic 1T phase formation. Second, heterojunction engineering enables the heterointerface to accelerate charge deployment and promote interface charge transfer. Third, the integration of WS_2_ and WSe_2_ leads to the redistribution of charge density at the heterointerface, which facilitates efficient electrocatalytic activity. The plasma-enhanced roughness of the WS_2_/WSe_2_ heterostructure has a fourth beneficial effect, enabling exposure of more active sites, which in turn promotes electron transfer between the electrocatalysts and electrolytes. Finally, the connection of WS_2_ with WSe_2_ creates a synergistic effect that incorporates the intrinsic characteristics of both components, shortening the electron transfer channels and enhancing the HER performance and stability.

## Conclusions

In summary, a novel synthesis of the WS_2_/WSe_2_ heterostructure was demonstrated by using a PACVR system. A metallic WS_2_/WSe_2_ heterostructure was synthesized at 350 °C, whereas a semiconducting heterostructure can be obtained at a higher temperature. Systematic characterizations revealed that the diffusion of Se atoms into the WS_2_ film leads to low reaction energy barriers by facilitating orbital overlap in the HER mechanisms. Interfacial electron transfer was observed in the WS_2_/WSe_2_ heterostructure, leading to the reduction of hydrogen atoms and an increase in the absorption capacity of the electrode surface. This resulted in a significant improvement in the kinetics of the HER. The WS_2_/WSe_2_ heterostructure fabricated by using this methodology exhibited excellent catalytic activity, with a low Tafel slope of 57 mV dec^–1^ and an overpotential of 291 mV at 10 mA cm^–2^. The electrochemically active surface area of the heterostructure confirmed its ability to improve electrocatalytic behavior. The surface electron conductivity was significantly improved due to the highly interacting interfaces in the heterostructure. The findings of this study offer a potential solution that is highly sought-after in the field of sustainable energy conversion technologies.

## Experimental Section

### Synthesis of the WS_2_/WSe_2_ Heterostructure Film

To prepare the WS_2_/WSe_2_ heterostructure, a WO_*x*_ seed layer was initially deposited on silicon wafers with a 300 nm thick SiO_2_ buffer layer under high vacuum (>10^–5^ Torr) conditions, through an E-gun evaporator system (a deposition rate of 0.1 Å s^–1^). This was followed by a two-step plasma-assisted chemical vapor reaction with the optimized parameters. First, the WO_*x*_-deposited wafers were transferred into the PACVR for the step of sulfurization. During the reaction, the sulfur precursor was vaporized at 180 °C, and the vapor was carried by a N_2_/H_2_ forming gas to reach the WO_*x*_-deposited substrate on a stage at a fixed elevated temperature of 350 °C through an ICP coil with a plasma of 150 W. Both the reduction of WO_*x*_ by hydrogen radicals and the chemical reaction between S radicals and W atoms were activated simultaneously, resulting in WS_2_ films. Subsequently, the sulfurized sample was quickly transferred to the selenization PACVR furnace for the conversion of the top layers of WS_2_ into WSe_2_. The synthesis process of the WS_2_/WSe_2_ heterostructure involved heating selenium pellets to 300 °C while maintaining the substrate at a low temperature of 350 °C in the presence of a N_2_/H_2_ atmosphere. This process was carried out for 1 h.

### Material Characterization

Various techniques were employed to characterize the synthesized films, as the presence of atom mixing, diffusion across interfaces, and precipitation of nanoparticles, which could affect the hydrogen uptake and release cycling, as well as the electronic and thermodynamic properties of the films.^54^ Raman spectroscopy using a 532 nm laser was used to investigate the uniformity and structure of the films, with the laser power set at 20 mW and the Si peak at 520 cm^–1^ serving as the standard peak. X-ray photoelectron spectroscopy (XPS) was utilized to analyze the binding energies and chemical compositions of the heterostructures using monochromatized Al Kα X-rays (*h*ν = 1486.6 eV) with an ECSA ULVAC-PHI 5000 Versaprobe II. The atomic geometry of the films was determined by cross-sectional transmission electron microscopy (TEM) using a JEOL JEM-200F with an acceleration voltage of 200 keV, and high-resolution TEM (HR-TEM) specimens were prepared using a focused ion beam instrument (FIB) (SII nanotech SMI3050) with the lift-out technique. The thickness, density, and roughness of each layer, as well as the heterostructure, were analyzed using X-ray reflectivity (XRR) on an eight-circle Huber diffractometer at the wiggler beamline BL17B at the National Synchrotron Radiation Research Center (NSSRC) in Taiwan, with GenX3 software used for data analysis. To study the depth of Se diffusion and interface characteristics, depth-spectrum XPS was conducted. Atomic force microscopy (AFM) (Bruker, dimension Icon) was used to examine the surface morphology and thickness of the films.

### Electrochemical Measurements

A conducting surface is required for electrochemical measurements. The as-synthesized WS_2_/WSe_2_ heterostructure films were transferred onto a gold-coated silicon substrate. To prepare the conducting substrate, a 300 nm SiO_2_/Si substrate was cleaned with acetone, isopropyl alcohol (IPA), and DI water, followed by the deposition of a 50 nm gold (Au) layer using an E-gun evaporation system under a high vacuum at a rate of 0.2–0.3 Å s^–1^. The synthesized film was separated from the Si surface using a PMMA layer as the support. The PMMA/2D material film was then removed from the pristine substrate by scraping its edges with tweezers. Between the PMMA/2D material film and the substrate, a space was created, and then it was transferred to a diluted ammonia solution (NH_4_OH/DI water = 1:5) for separation and finally peeled off in DI water for cleaning before being transferred onto the conducting Au/Si substrate. The whole transferred film Au/Si substrate was kept in an acetone solution, followed by cleaning with IPA and DI water to dissolve the PMMA. The electrochemical characteristics of the samples were assessed by using a three-electrode configuration and a Bio-Logic VSP potentiostat inside a Teflon cylinder cell equipped with an O-ring at the base. The reference and counter electrodes used were Ag/AgCl saturated with 3 M NaCl and a Pt wire, respectively. The electrolyte solution used for all electrochemical measurements was 0.5 M H_2_SO_4_. The WS_2_/WSe_2_ heterostructure film was transferred to a 50 nm thick Au-deposited Si substrate and used as a catalyst for the hydrogen evolution reaction, with Cu tape (3 M) for connection. The measurements were recorded after ten cycles without magnetically stirring the solution. Polarization curves were obtained by conducting linear sweep voltammetry (LSV) at a scan rate of 5 mV/s. All potentials were calibrated to potentials versus a reversible hydrogen electrode (RHE) with reference to Ag/AgCl using the Nernst equation given by *E*_RHE_ = *E*_Ag/AgCl_ + 0.0591pH + *E*°_Ag/AgCl_. The correction for internal resistance (IR) was performed in order to adjust for the potential drop losses that occur due to the resistance of the solution. This correction is carried out using the equation given by *E*_corrected_ = *E*_RHE_ – *iR*_S_. The Tafel plots, which show the relationship between the overpotential and the logarithm of current density, were analyzed by fitting them to a linear equation. The linear slope was then determined as the Tafel slope using the provided formula, η = *a* + *b  *log*  j*, for which η is the overpotential, *j* is the current density, *b* is the Tafel slope, and *a* is the intercept. The long-term stability was obtained at a 6 mV constant potential from the chronoamperometry test by recording the current versus time. In order to calculate the electrocatalytically active surface area (ECSA), cyclic voltammetry (CV) was performed in the non-faradaic reaction potential range at different scan rates. The double-layer capacitance was derived from the CV data and used in the following equation given by ECSA = *C*_dl_/*C*_s_, for which *C*_s_ is the specific capacitance. The Nyquist plot was obtained by conducting electrochemical impedance spectroscopy (EIS) measurements in the frequency range of 0.1 Hz to 100 kHz at an amplitude of 10 mV.

## Supporting Information Available

Schematic process flows of the WS_2_/WSe_2_ film transfer on conducting the Au substrate for device formation; three-electrode system for electrochemical measurements using a Bio-Logic VSP potentiostat in a cylindrical cell made of Teflon with an O-ring at the bottom; Raman spectra for WS_2_ and WS_2_/WSe_2_ growth on a sapphire substrate; Raman spectra of the MoS_2_/MoSe_2_ heterostructure with MoS_2_ and MoSe_2_; XPS spectra: (a) survey of WO_*x*_, (b) survey spectra of WS_2_, (c) survey spectra of WSe_2_, (d) O 1s binding energy for the WS_2_/WSe_2_ heterostructure, WS_2_, and WO_*x*_; XPS spectrum analysis of the MoS_2_/MoSe_2_ heterostructure; TEM analysis: WO_*x*_ and WS_2_; high-resolution TEM images; energy-dispersive spectroscopy (EDS) elemental mapping of each element, respectively; Raman spectra of the WS_2_/WSe_2_ heterostructure after HER; equivalent circuit model for EIS analysis of the catalysts; ECSA-normalized LSV curves; electrocatalytic performance of different catalysts for HER in 0.5 M KOH; turnover frequency (TOF) calculations, and effect of component ratios on HER performance (PDF)
