# Supplementary material for: Rational Design of Phase-Engineered WS2/WSe2 Heterostructures by Low-Temperature Plasma-Assisted Sulfurization and Selenization toward Enhanced HER Performance
Source: ACS Appl Mater Interfaces. 2024 Jun 11;16(25):32490–502. doi: 10.1021/acsami.4c03513 (PMC11212026; doi:10.1021/acsami.4c03513)
Supplement: Supplementary file 1 — am4c03513_si_001.pdf [file am4c03513_si_001.pdf]

Supporting information

Rational Design of Phase-Engineered WS_2/WSe_2 Heterostructures by Low-Temperature Plasma-Assisted Sulphurization and Selenization Towards Enhanced HER Performance

Bushra Rehman[1,2,3], K.M.M.D.K. Kimbulapitiya[1,2,3], Manisha Date[1,2,3], Chieh-Ting Chen[1,2,3], Ruei-Hong-Cyu[1,2,3], Yu-Ren Peng[1,2,3], Mayur Chaudhary[1,2,3], Feng-Chuan Chuang[4], and Yu-Lun Chueh[1,2,3,4]*

[1]*Department of Materials Science and Engineering, National Tsing Hua University, Hsinchu 30013, Taiwan*

[2]*College of Semiconductor Research, National Tsing Hua University, Hsinchu 30013, Taiwan*

[3]*Department of Physics, National Sun Yat-Sen University, Kaohsiung, 80424, Taiwan*

[4]Department of Materials Science and Engineering, Korea University, Seoul 02841, Republic of Korea.

* E-mail: ylchueh@mx.nthu.edu.tw

Table S1. The R_s value of as-synthesis catalyst.

Catalyst	WS_2	WSe_2	WS_2/WSe_2
R_s (Ohm)	14.8	13.6	12.6
R_{ct} (Ohm)	186.7	49.9	6.37

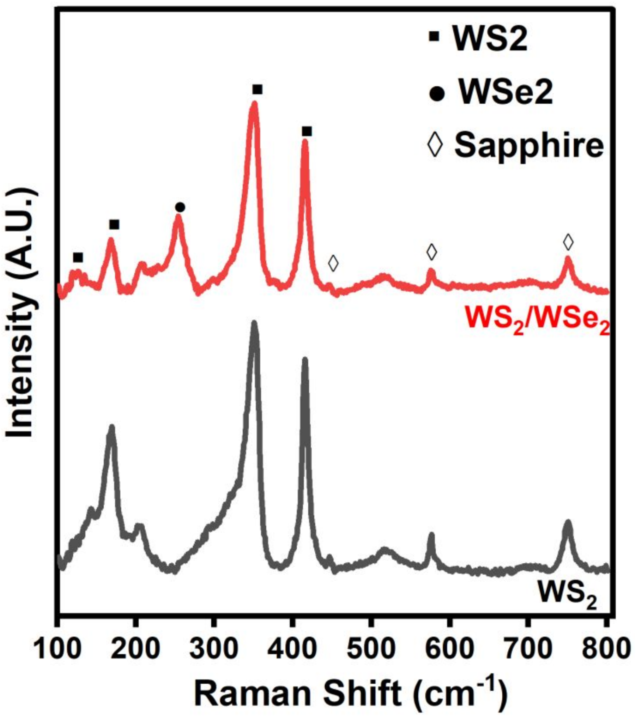

Figure S1. Raman spectra for WS_2 and WS_2/WSe_2 growth on a sapphire substrate

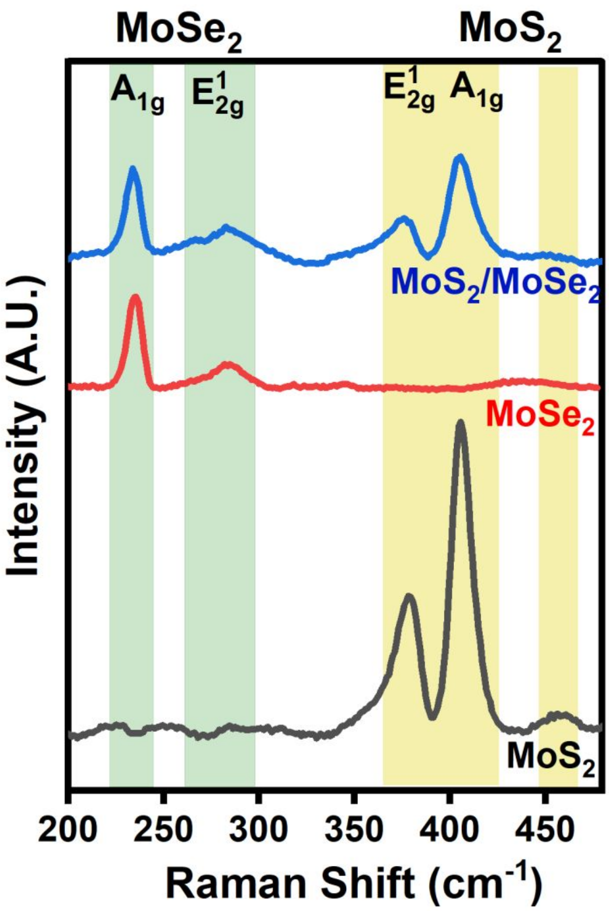

Figure S2. Raman Spectra of $MoS_2/MoSe_2$ heterostructure with MoS_2 and $MoSe_2$.

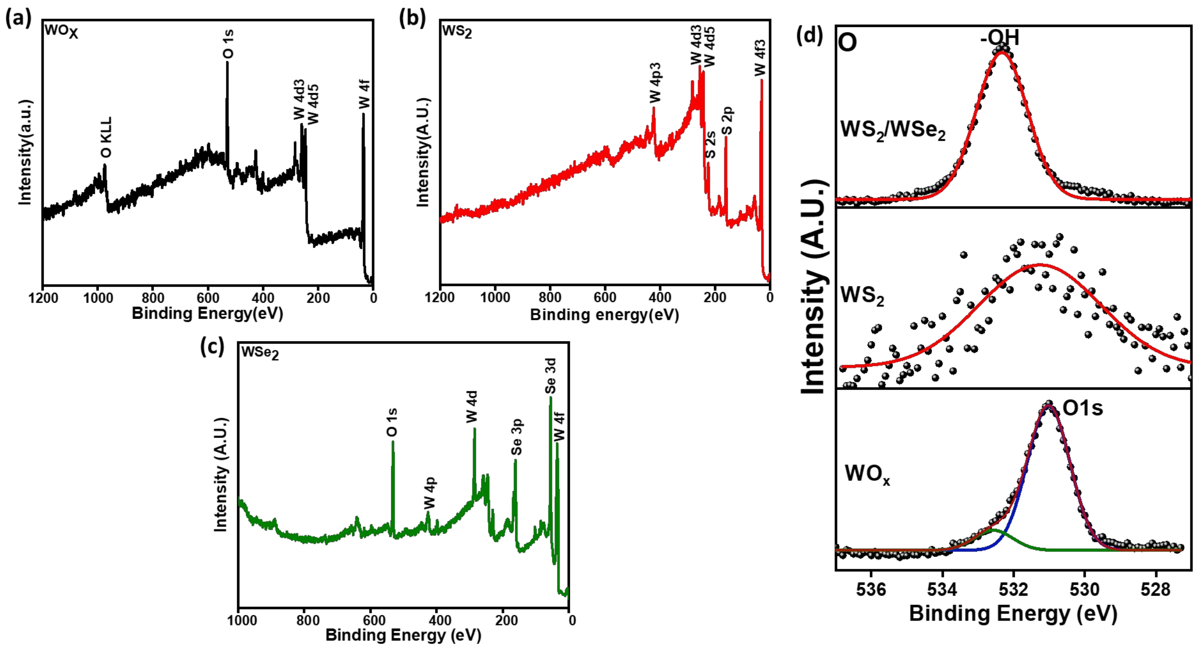

Figure S3. XPS spectra (a) Survey of WO_x, (b) Survey spectra of WS_2, (c) Survey spectra of WSe_2, (d) O 1s binding energy for WS_2/WSe_2 heterostructure, WS_2, and WO_x

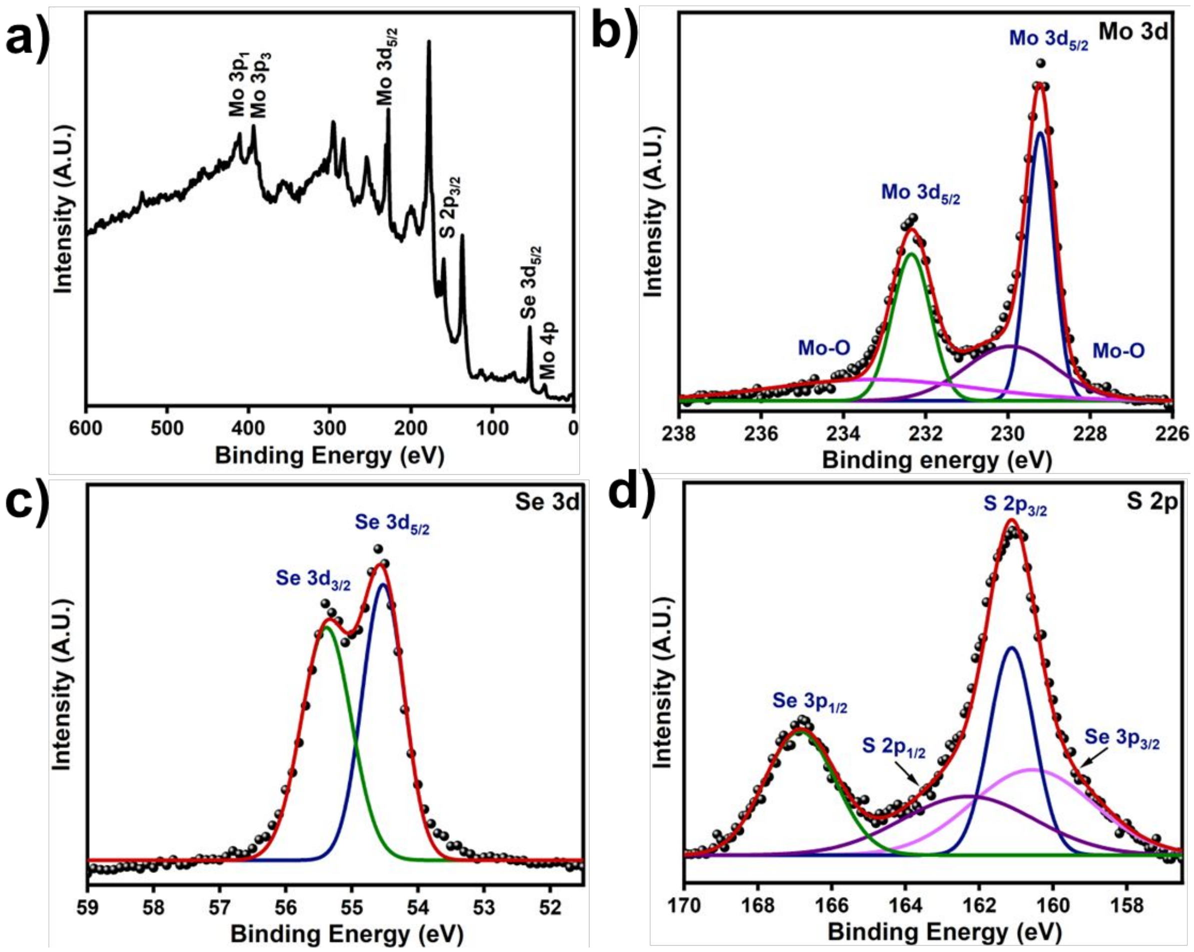

Figure S4. (a) Overall XPS spectrum analysis of the $MoS_2/MoSe_2$, (b) Mo 3d, (c) Se 3d, and (d) S 2p high-resolution XPS of $MoS_2/MoSe_2$ catalyst.

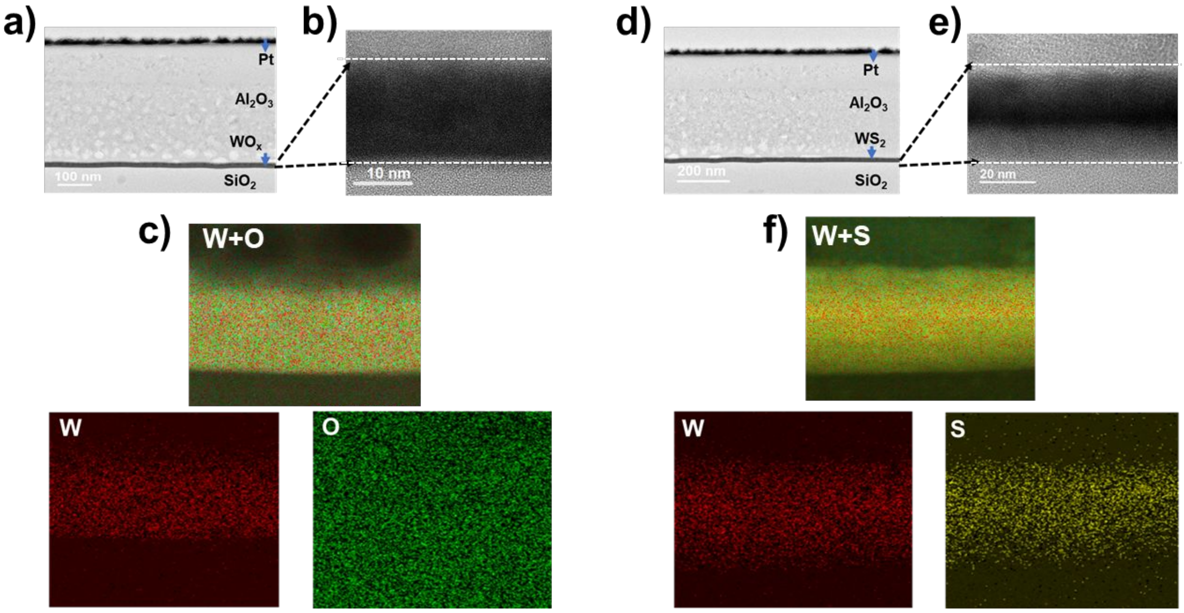

Figure S5. TEM Analysis WO_x and WS_2 (a, d) low magnification image (at the scale of 200 nm) (b, e) High-resolution TEM images (c, f) EDS elemental mapping of each element, respectively.

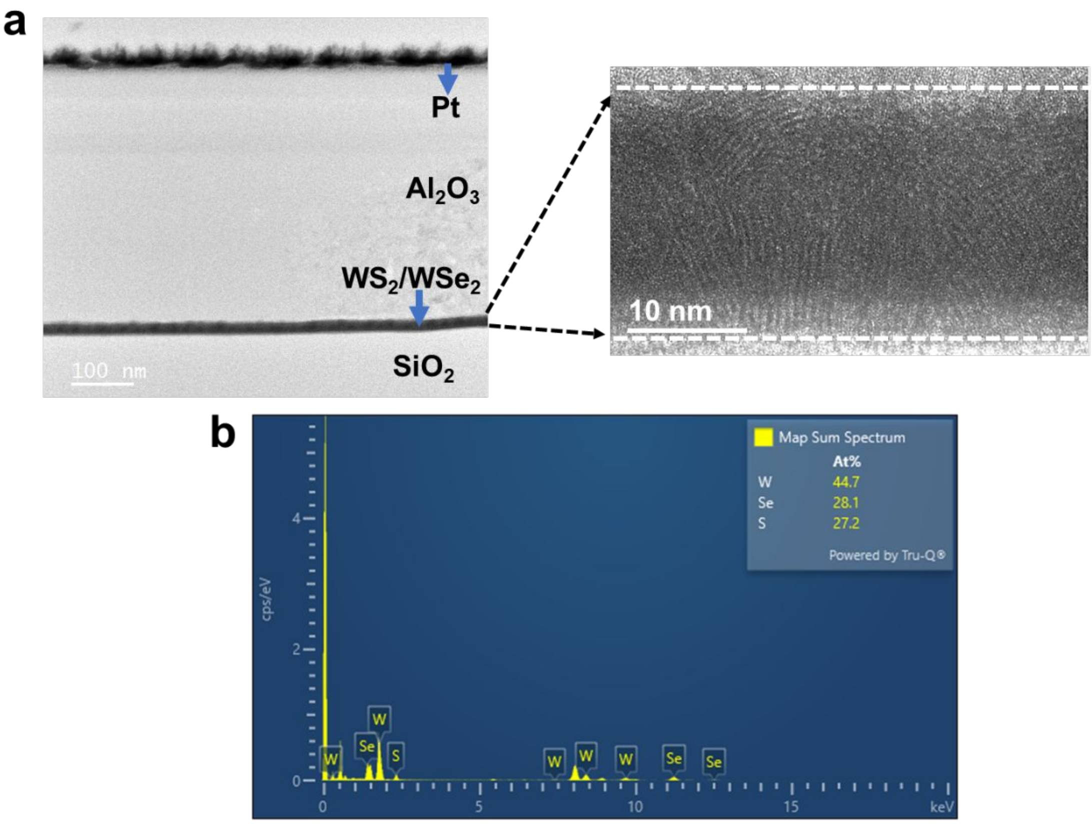

Figure S6. TEM Analysis of WS_2/WSe_2 heterostructure (a) low magnification image (at the scale of 100 nm) and High-resolution TEM image (b) EDX spectra.

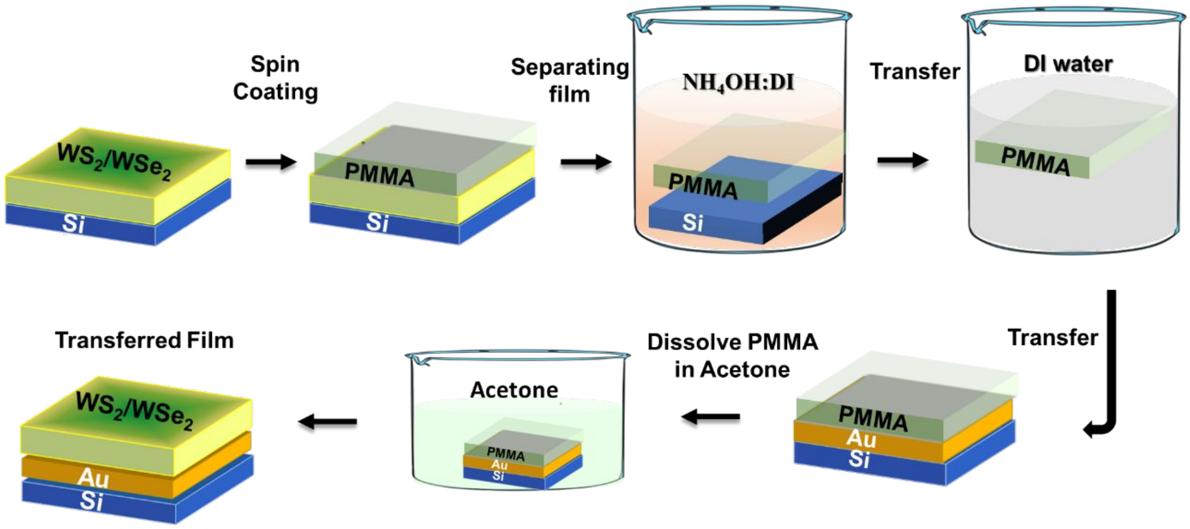

Figure S7. Schematic process flows of WS_2/WSe_2 film transfer on conducting Au substrate for device formation.

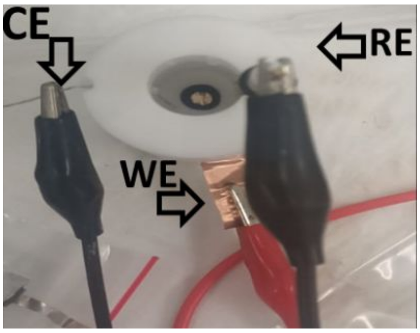

Figure S8. Three-electrode system for electrochemical measurements using a Bio-Logic VSP potentiostat in a cylindrical cell made of Teflon with an O-ring at the bottom. Ag/AgCl saturated with 3 M NaCl and a Pt wire were used as the reference and counter electrodes, respectively, in a 0.5 M H_2SO_4 electrolyte solution (where CE represents the counter electrode, RE is the Reference electrode and WE are the working electrode)

Turnover Frequency (TOF): TOF calculations were carried out using the equation from the previous reports[37]:

$$TOF = \frac{JA}{2Fn}$$

Where J represents the corresponding current density under a certain overpotential of the LSV result; A is the surface area of the electrode (0.212 cm^2); F represents the Faraday constant (96485.3 A mol^{-1}); n is the no. of moles at the covered electrode area.

Figure S9. shows the TOF performance of the WS_2/WSe_2 heterostructure at different overpotentials. The heterostructure exhibits a better TOF of 2.7 s^{-1} than pure WS_2 and WSe_2 whose calculated values are 0.4 s^{-1} and 0.89 s^{-1}, respectively, at an overpotential of 300 mV.

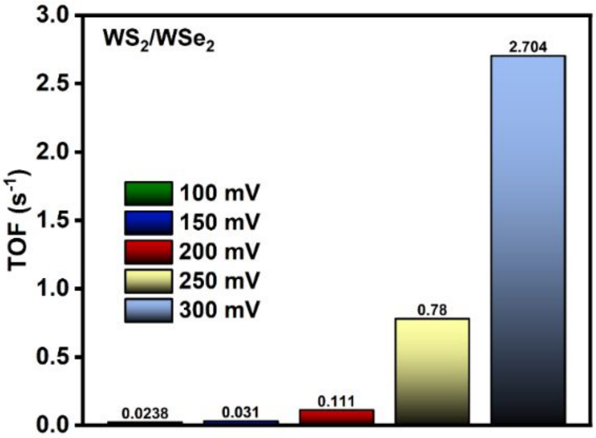

Figure S9. TOF values of WS_2/WSe_2 heterostructure measured at different overpotentials

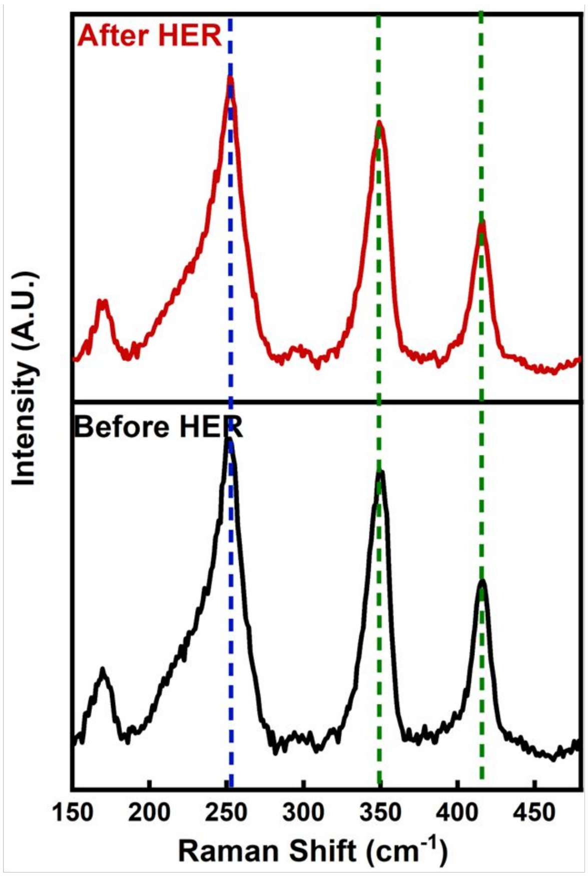

Figure S10. Raman Spectra of WS_2/WSe_2 heterostructure before and after HER

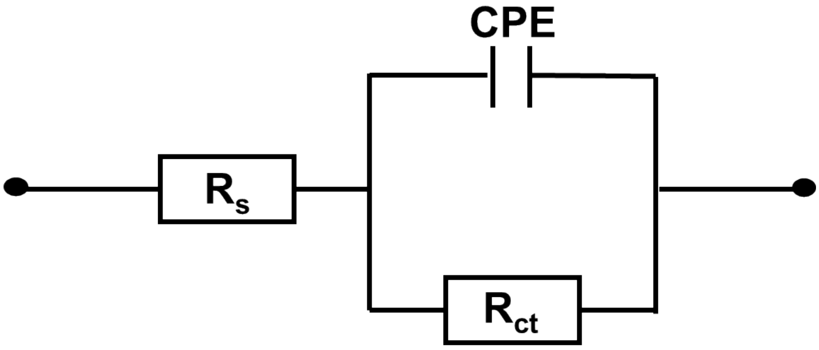

Figure S11. Equivalent circuit model for EIS analysis of the catalysts

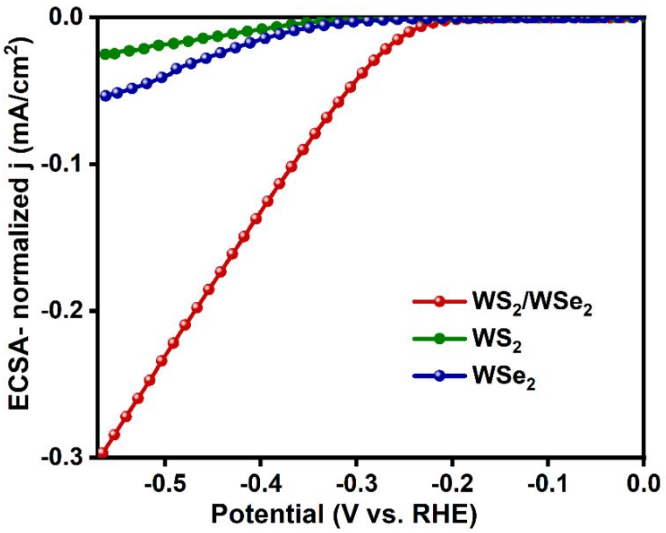

Figure S12 ECSA- normalized LSV curves

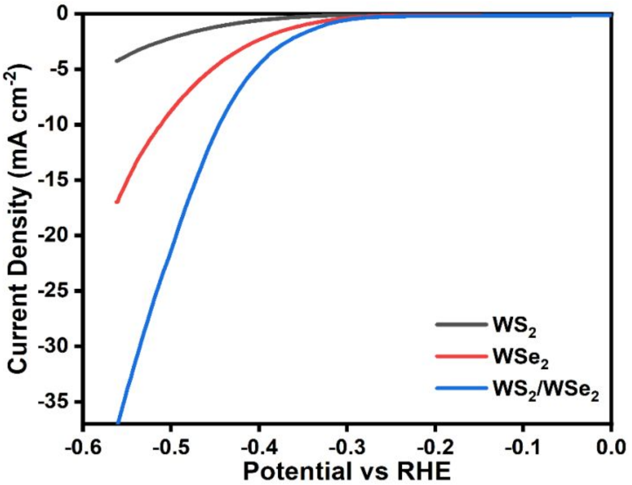

Figure S13. Electrocatalytic performance of different catalysts for HER in 0.5 M KOH

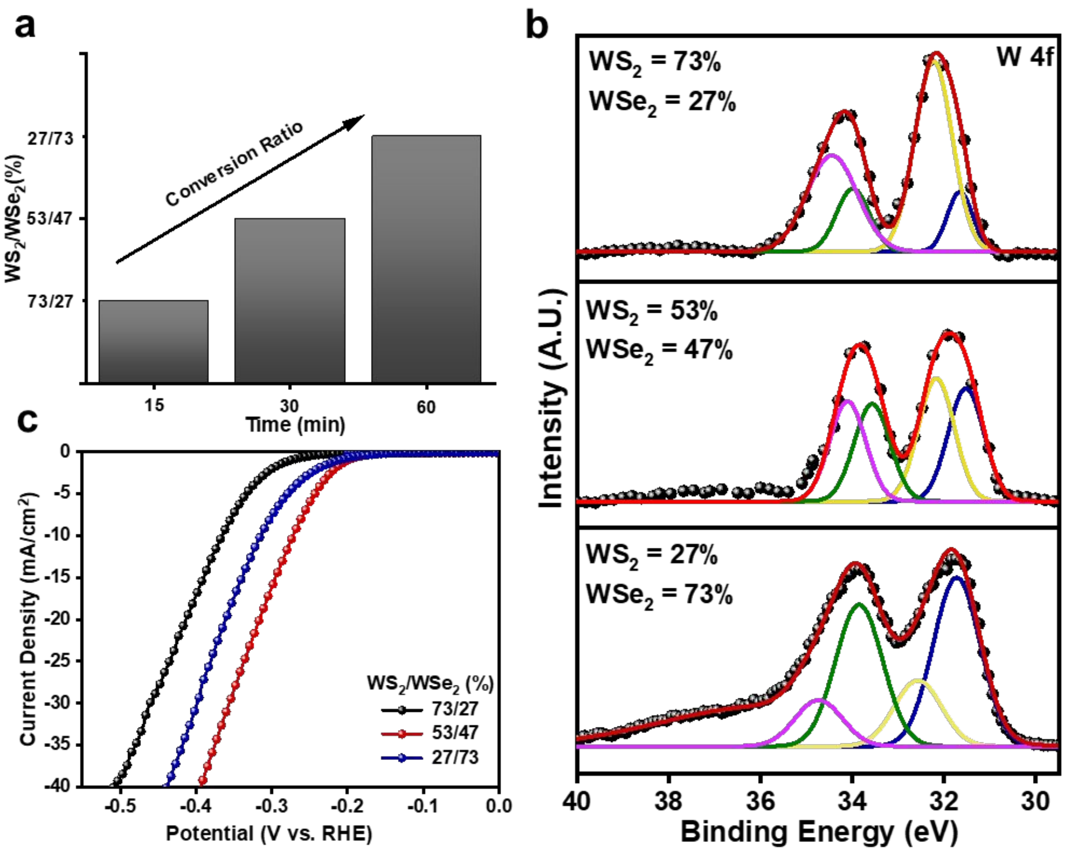

Figure S14. (a) Selenization conditions for 2D-layered WS_2 films exposed to the plasma power for different growth times at plasma-assisted selenization temperature of 350 ^oC. (b) The corresponding W4f spectra of the 2D-layered Heterostructured film. The plot shows the conversion of WS_2 films to WS_2/WSe_2 heterostructures as a function of growth time. (c) LSV curves for the different ratios of WS_2 and WSe_2 in the heterostructures.
